# Iatrogenic Kaposi sarcoma of the small bowel in Crohn’s disease following short-term use of immunomodulators: a case report and review of the literature

**DOI:** 10.1186/s13256-022-03637-8

**Published:** 2022-11-07

**Authors:** Pei-Jui Wu, Chi-Shu Sun, Hsing-Tao Kuo, Ming-Jen Sheu, Cheng-Yi Lin, Su-Hung Wang, Chun-Chi Yang, Chi‐Hsing Chen, Shih-Sung Chuang, I-Che Feng

**Affiliations:** 1 Division of Hepatogastroenterology, Department of Internal Medicine, Chi Mei Medical Center, No.901, Zhonghua Rd. Yongkang Dist., Tainan City, 71004 Taiwan, ROC; 2 Department of Pathology, Chi Mei Medical Center, Tainan, Taiwan, ROC; 3 College of Recreation and Management, Chia Nan University of Pharmacy and Science, Tainan, Taiwan, ROC

**Keywords:** Crohn’s disease, Chemotherapy, Gastrointestinal Kaposi sarcoma, Immunomodulator, Kaposi sarcoma

## Abstract

**Background:**

Kaposi sarcoma is a vascular tumor highly related to human herpesvirus-8 and Kaposi sarcoma–associated herpesvirus. Kaposi sarcoma usually manifests as skin or mucosal lesions; involvement in visceral organs such as the gastrointestinal tract is rare. Kaposi sarcoma can occur in immunocompromised patients receiving immunosuppressive therapy, in which case it is known as iatrogenic Kaposi sarcoma or drug-induced Kaposi sarcoma. Intestinal Kaposi sarcoma in patients with inflammatory bowel disease is extremely rare.

**Case presentation:**

A 46-year-old East Asian male with recently diagnosed Crohn’s disease was administered azathioprine and prednisolone; however, the patient complained of persistent abdominal pain and diarrhea following treatment. Endoscopy revealed small bowel Kaposi sarcoma. The patient was treated with systemic chemotherapy successfully without relapse.

**Conclusions:**

This is the fifth case of Kaposi sarcoma developed over the small intestine in a patient with Crohn’s disease following administration of immunomodulators. Additionally, this case indicated that even short-term immunomodulator use can induce Kaposi sarcoma in patients with inflammatory bowel disease. Thus, in patients with inflammatory bowel disease, if symptoms are aggravated or do not abate after immunomodulators prescription, and before intending to upgrade immunomodulators, endoscopy should be considered. Finally, chemotherapy can also be considered if both medication withdrawal and surgical intervention are not feasible.

## Background

Kaposi sarcoma is an opportunistic, angioproliferative tumor, closely related to human herpesvirus-8 (HHV-8) and Kaposi sarcoma–associated herpesvirus (KSHV). In addition to widely recognized cutaneous or mucosal manifestations, visceral involvement has been described. KS that occurs in immunocompromised patients receiving immunosuppressive therapy, not including KS that is associated with human immunodeficiency virus (HIV), is known as iatrogenic KS or drug-induced KS [1, 2].

Inflammatory bowel disease (IBD) is a condition of chronic inflammation of the gastrointestinal tract. Patients with IBD usually require one or more immunomodulators if they are resistant to basic treatment. Intestinal KS following immunomodulation in patients with IBD [3–6] is a rare, iatrogenic complication. Furthermore, KS that occurs in the small intestine is extremely rare, especially as a result of the interaction between Crohn’s disease and the use of immunomodulators.

Here, we report a case of KS that developed in the small intestine in a patient with Crohn’s disease following short-term administration of immunomodulators.

## Case presentation

A 46-year-old East Asian male with a history of Crohn’s disease (diagnosed in November 2018 with duodenitis, duodenal ulcers, ileitis, and colitis with crypt abscess) reported to have no psychosocial disorder nor significant family history. He was treated with immunomodulators including an initial treatment of azathioprine 50 mg and prednisolone 15 mg twice daily, and mesalazine. Owing to remission of the clinical symptoms of Crohn’s disease, prednisolone was gradually tapered off; prednisone use covered a total period of 3 months.

**Fig. 1:**
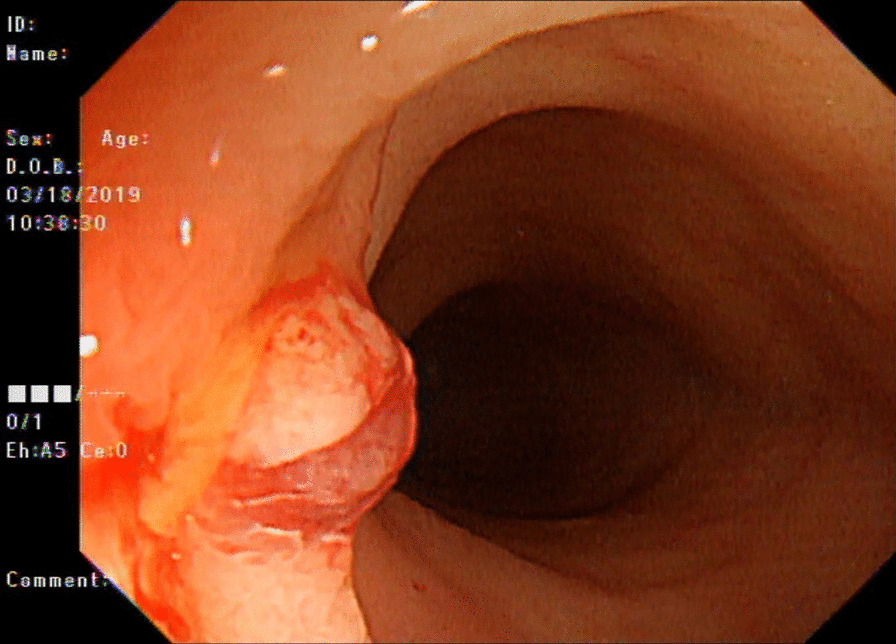
Endoscopy showed polypoid lesion of the terminal ileum. Biopsy of the polypoid tumor and histopathology identified Kaposi sarcoma

**Fig. 2:**
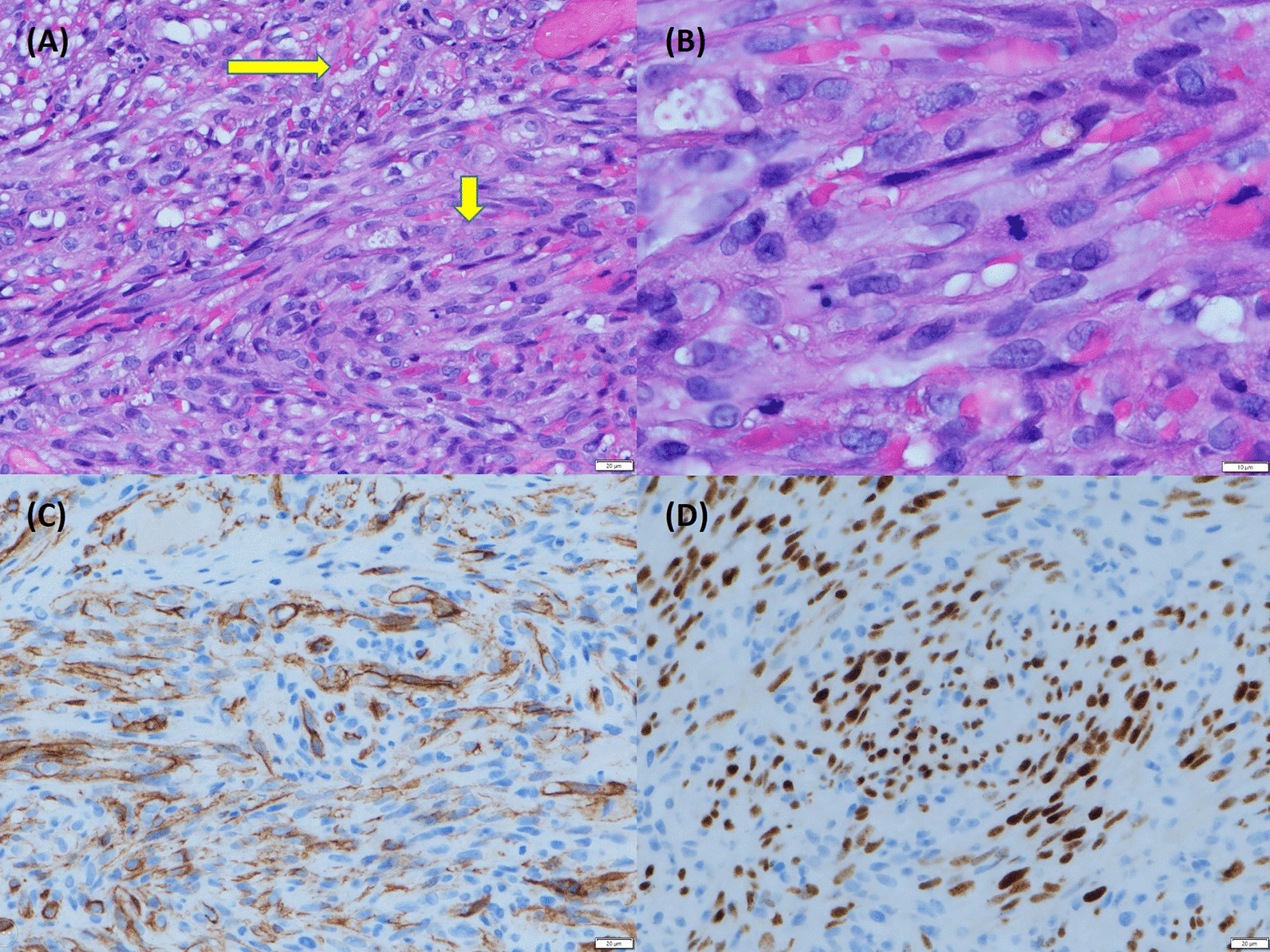
Histopathology of Kaposi sarcoma in the terminal ileum. Spindle cells were arranged in intersecting fascicles with intervening slit-like vascular spaces containing erythrocytes located in the submucosal layer. **A** Fascicles of spindle cells (short arrow) and extravasated erythrocytes (long arrow) on Hematoxylin and Eosin stain (× 400). **B** Spindle cells on Hematoxylin and Eosin stain (× 1000). **C** Immunohistochemical staining for CD 34 was positive (× 400). **D** Immunohistochemical staining for HHV-8 was positive (× 400)

Following 4 months of azathioprine and 3 months of prednisolone use, the patient complained of persistent abdominal pain and diarrhea. Endoscopy in March 2019 revealed scarring in the colon and terminal ileum. Furthermore, inflammatory polypoid lesions were noted in the terminal ileum that were not present in a previous examination (Fig. 1). Results of a biopsy revealed that spindle cells were arranged in intersecting fascicles with intervening slit-like vascular spaces containing erythrocytes located in the submucosal layer. Immnunohistochemistry revealed immunoreactivity for CD34, smooth muscle actin (SMA), and HHV-8. Stainings for CD117, DOG1, and cytomegalovirus were all negative (Fig. 2). These findings supported a diagnosis of KS and excluded a diagnosis of gastrointestinal stromal tumor.

**Fig. 3:**
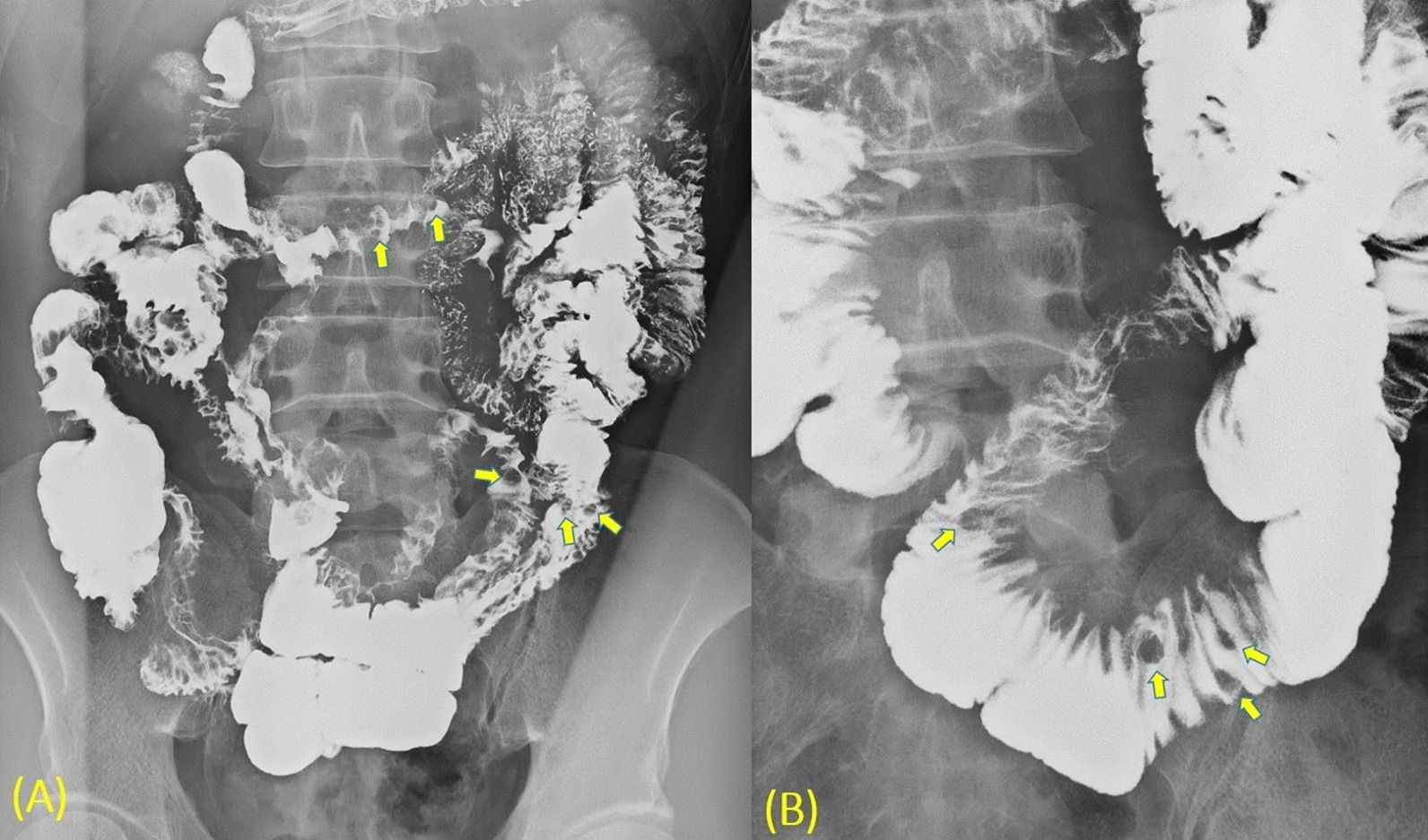
Small bowel series indicated (**A**) Multiple smooth-surface round filling defects throughout the jejunum to the ileum (arrows) (**B**) Multiple smooth-surface round filling defects over the ileum, compatible with polypoid tumors (arrows)

**Fig. 4:**
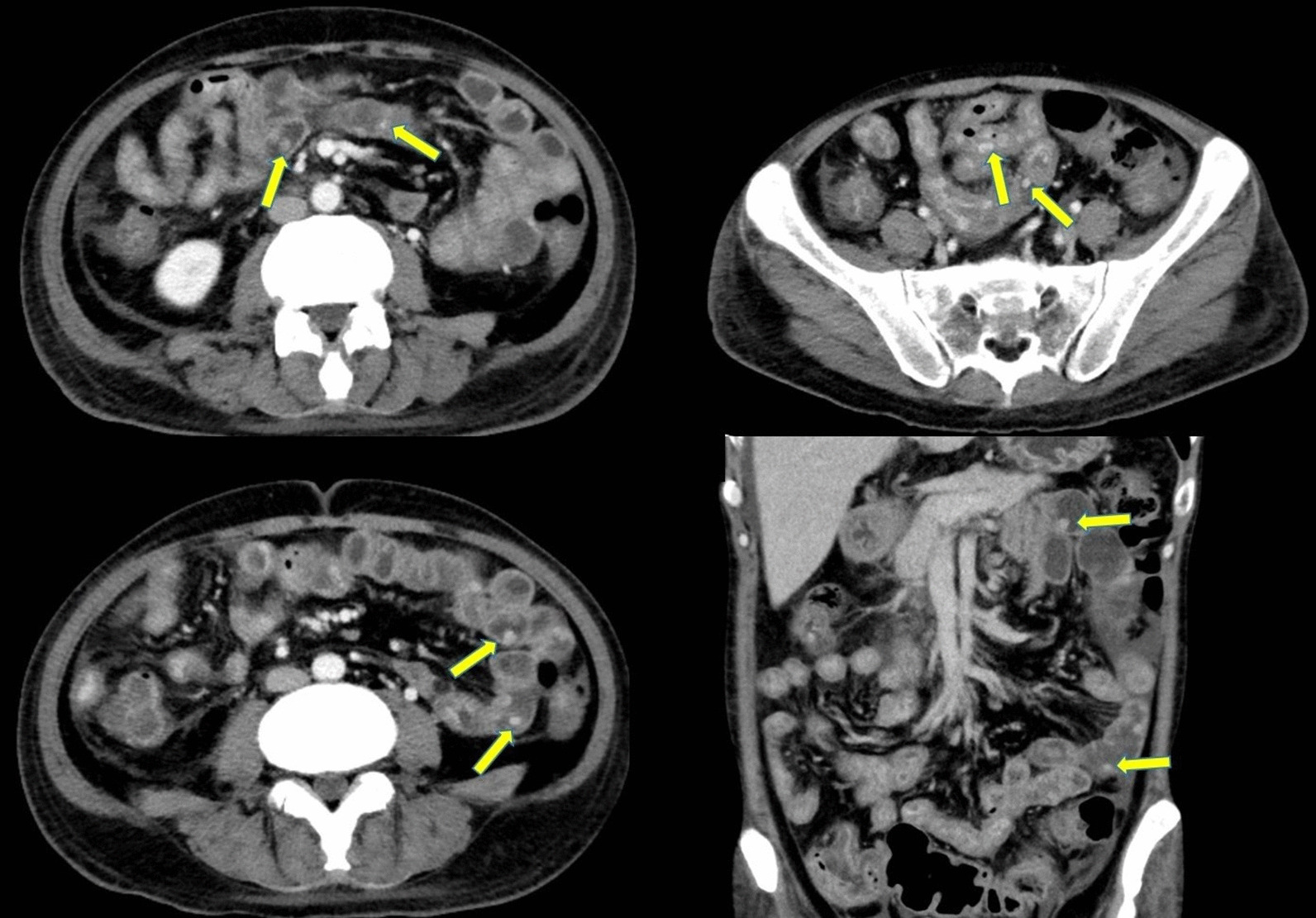
Abdominal CT scan revealed diffuse edematous change of gastrointestinal tract and multiple hyperdense nodular lesions in the small bowel (arrows)

The patient was tested for HIV, hepatitis C virus, and herpes simplex virus, with negative results. Physical examination revealed no cutaneous manifestations of KS. Small bowel series indicated multiple smooth-surface round filling defects throughout the jejunum to the ileum, compatible with intraluminal polypoid tumors (Fig. 3). Abdominal computed tomography (CT) revealed diffuse edematous change of the gastrointestinal tract and multiple nodular lesions in the small bowel (Fig. 4). Therefore, we reduced administration of azathioprine 50 mg from twice to once daily and reintroduced prednisolone to control the symptoms of Crohn’s disease.

The oncologist began chemotherapy with eight courses of epirubicin 100 mg. After 6 months, endoscopy and CT revealed complete remission of KS, and the symptoms of Crohn’s disease were stable. During chemotherapy, doses of the aforementioned immunomodulators were kept constant, and doses of prednisolone were adjusted ranged from 2.5 mg twice daily to 10 mg twice daily according to variations in the clinical activity of the patient’s Crohn’s disease. Following chemotherapy, the patient was monitored with CT every 4 months in the first year and every 6 months in subsequent years. Magnetic resonance (MR) enterography and endoscopy were used when symptoms of Crohn’s disease flared up. The patient did not relapse for more than 2 years.

## Discussion and conclusions

Iatrogenic KS is a variant of KS that is associated with immunosuppression or transplantation that develops after immunosuppressive therapy. Classic KS presents predominantly with multiple vascular cutaneous and mucosal nodules, whereas iatrogenic KS is more likely to involve the lymph nodes, mucosa, and visceral organs, including of those of the gastrointestinal tract, and sometimes absence of manifestation in the skin [2, 7]. KS-associated gastrointestinal lesions are rare. Gastrointestinal lesions usually lack symptoms, but can cause bleeding or lead to an obstruction [1, 8, 9]. Therefore, intestinal KS is usually detected using endoscopy and confirmed with histopathology, as was the case with this patient.

Given that intestinal KS is located in the submucosa with intraluminal growths, endoscopy typically reveals an intraluminal nodular or polypoid tumor with a reddish, violaceous brown or blue color [4, 5]. Pseudopolyps (secondary to severe Crohn’s disease) and vascular malignant tumors such as angiosarcoma should also be considered [6]. Histopathology is the gold standard in the diagnosis of intestinal KS, but we should also consider the possibility of a biopsy returning a false negative owing to the challenges inherent in sampling submucosal lesion [5]. The histopathology f KS typically presents as spindle cells proliferation, aggregates as fascicles, and is separated by slit-like spaces. Extravasated erythrocytes, siderophages, inflammatory lymphocytic and plasma cells are usually present between the spindle cells [1, 10]. A KS spindle cells stain may be positive for vascular markers such as ERG, CD34, and CD31 [10].

There are no specific staging systems or treatment guidelines for iatrogenic KS [1]. Present consensus suggests spontaneous remission of iatrogenic KS occurs after discontinuation of or a switch to another immunosuppressive therapy in patients who have undergone an organ transplant [1, 3, 4, 7, 11]. Unfortunately, withdrawal or a reduction in the dose of immunomodulators is difficult to implement in some situations (for example, in patients with IBD that frequently flares up). Consequently, surgical excision, such as small bowel resection or colectomy, is an alternative treatment option.

Intestinal KS in patients with IBD following administration of immunomodulators is extremely rare [3–5]. We searched the Pubmed database using combinations of the terms “Crohn’s disease,” “ulcerative colitis,” and “Kaposi sarcoma” and found only case reports on this issue. Intestinal KS was mentioned in most of the cases involving ulcerative colitis but in only five cases involving Crohn's disease, four of which involved the small bowel (Table 1), out of the total of Pubmed’s English-language literature [5, 6]. Our patient was the fifth case of KS in the small intestine in a patient with Crohn's disease.

**Table 1:** Intestinal Kaposi sarcoma in HIV-negative patients with Crohn’s disease in the literature

First author, reported year, and journal	Age (years)/sex	Immunomodulators for Crohn’s disease	Duration of developed KS after immunomodulators	KS location	Skin lesions	HHV-8 status	Treatment of KS
Koop, 1987	29/F	Prednisone	Long-standing	Jejunum, bowel mesentery	Yes	NA	Small bowel resection
*Am J Med*							
Puy-Montbrun, 1991	36/F	Steroids	54 months	Colon	No	NA	Immunomodulators withdrawal
*Dig Dis Sci*		Azathioprine	11 months				
Cohen, 2001	67/F	Prednisone	Long-standing	Ileum, colon	No	NA	Ileocolic resection
*Am J Gastroenterol*							
Annika, 2017	21/M	Prednisone	Long-standing	Terminal ileum	No	Negative	Immunomodulators withdrawal
*Int. J. Surg. Pathol*		Infliximab	3 doses				
Elisa Stasi, 2019	45/F	Steroids	First 3 months	Terminal ileum	NA	Positive	Ileocecal resection
*Medicine*		Infliximab	9 months after administration				
Present case	46/M	Prednisone	3 months	Small bowel	No	Positive	Chemotherapy
		Azathioprine	4 months				

*F* female, *HIV* human immunodeficiency virus, *HHV-8* human herpesvirus-8, *M* male, *NA* no available data, *KS* Kaposi sarcoma

We found the present case worthy of discussion. Firstly, the patient received a combination of the immunomodulators azathioprine and prednisolone for 3 months and then azathioprine alone for 1 month. The period of exposure to the immunomodulators prior to the development of KS was relatively short. By contrast, the average period of exposure to immunomodulators in the cases in the literature of patients with KS and IBD ranged from 1 to 2 years. A few cases of patients with ulcerative colitis reported administration of immunomodulators for just 1–3 months prior to the development of intestinal KS [3, 5]. We believe that even short-term exposure to immunomodulators has the potential to induce KS. Secondly, the previous cases mostly chose to manage iatrogenic KS using enterectomy or withdrawal of immunomodulators. In our case, neither of those two options were feasible. Thus, owing to a lack of treatment guidelines, we chose chemotherapy. For the management of other variants of KS, systemic chemotherapy can be applied in aggressive or disseminated cases or those with symptomatic visceral involvement [9, 11, 12]. Liposomal anthracyclines or taxanes are the first line of single-agent chemotherapy [9, 12]. Our patient is the first patient with intestinal KS and Crohn’s disease to be treated with chemotherapy. Thirdly, there is still no consensus on the use of immunomodulators or biological drugs during chemotherapy in IBD patients and more investigations are needed [13, 14]. In our case, we chose to reduce the doses of immunomodulators and adjusted the doses of prednisolone according to clinical activity of the patient’s Crohn’s disease during chemotherapy. Our patient did not relapse in the 2-year observational period following concomitant use of immunomodulators and chemotherapy.

In summary, we reported a case of isolated intestinal KS, a rare complication of IBD, that manifested as submucosal polypoid lesions that was diagnosed using endoscopy. Histopathology was required for a definite diagnosis and to exclude other diseases. Additionally, this case indicated the possibility that even short-term immunomodulators can induce KS in patients with IBD. Thus, in patients with IBD, if symptoms are aggravated or do not abate following immunomodulator administration and before we intended to upgrade immunomodulators, endoscopy should be considered. Finally, chemotherapy could also be considered if medication withdrawal and surgical intervention are not feasible.

## Data Availability

Data supporting the case report may be provided by the authors upon reasonable request.

## Abbreviations

CT: Computed tomography
HIV: Human immunodeficiency virus
HHV-8: Human herpesvirus-8
IBD: Inflammatory bowel disease
KS: Kaposi sarcoma
KSHV: Kaposi sarcoma–associated herpesvirus
MR: Magnetic resonance
SMA: Smooth muscle actin
